# Supplementary material for: Dynamics in the Sakaguchi-Kuramoto model with bimodal frequency distribution
Source: PLoS One. 2020 Dec 9;15(12):e0243196. doi: 10.1371/journal.pone.0243196 (PMC7725404; doi:10.1371/journal.pone.0243196)
Supplement: Attachment — Submitted filename: Response to Reviewers.doc [file pone.0243196.s001.doc]

**Manuscript No.:** PONE-D-20-12498

**Title:** Dynamics in the Sakaguchi-Kuramoto Model with bimodal frequency distribution

Dear Editor,

Thank you very much for handling our manuscript. We really appreciate the insightful comments from the Referee. We have revised the manuscript based on these comments. We hope this revised version could meet the publication standard of PLOS ONE.

For the revised submission, we would like to update our Funding Statement as follows: “This work is supported by National Natural Science Foundation of China under Grants No. 11575036 and No. 11805021, and BUPT Excellent Ph.D. Students Foundation under Grant No. CX2019138. The funders had no role in study design, data collection and analysis, decision to publish, or preparation of the manuscript.”

In the initial submission, we provided the funding statement in the manuscript but did not state them in the Funding section of the online submission. We must apologize for our carelessness. We would be grateful if you could help us update the financial disclosure statement.

**The followings are the details of the reply.**

**Reviewer #1:** The authors study and report the bifurcation structure of the Sakaguchi-Kuramoto model with bimodal distribution. The work is a natural extension of Martens et al., PRE 2009 that allows for asymmetric solutions and includes a phase shift parameter \beta.

The first set of results are obtained by increasing \beta from zero, and examining the nature of the equilibrium and limit cycle solutions. In particular, the changes that occur in the two-dimensional bifurcation diagram are presented.

The second set of results involve the "revival" of the incoherent state. The authors consider various ways to break the symmetry of the bimodal natural frequency distribution. They find parameter space regions where the stability of the incoherent state goes from stable to unstable to stable again by varying one parameter.

In general, the work appears to be largely correct and interesting. However, there are a few problems with citations and terminology, and the exposition of the results section should be improved.

**Comment 1:** First, this work is so closely related to Martens et al. (2009) -- Ref. 35 in the manuscript -- that it should be placed explicitly in that context. Specifically, Martens et al. (2009) should be introduced and cited by name in the Introduction of this manuscript (the way that many other references are cited there). The results of Martens et al. (2009) should be discussed, and the relationship of the authors' work to that reference described.

**Reply:** Thank you very much for the suggestions. We have mentioned the work of Martens et al. (2009) in the introduction section and added several sentences to discuss the results of them (lines 50-54). We also clarified the relationship of our work to the reference in the main text (lines 61-62, 180-182).

**Comment 2:** Second, the authors refer to pitchfork bifurcations of the incoherent state (i.e., the origin). Pitchfork bifurcations involve one equilibrium transitioning into three with a change of stability of the original equilibrium. In the system studied, the origin (which has pairs of degenerate eigenvalues) transitions into two equilibria. The authors mention that the apparently missing equilibrium corresponds to an "unrealistic" solution with a negative values of the absolute values of z1 and z2.
The authors also claim that other publications (their refs. 35 and 36) that have identified these bifurcations of the incoherent state as (degenerate) transcritical bifurcations are wrong. If the authors want to say this, then they need to establish their claim by providing the relevant details. If I remember correctly, one way to view the bifurcation of the incoherent state is to consider two coincident equilibria existing at the origin (due to the degeneracy). At the transcritical bifurcation, these exchange stability and the stable one migrates away from the origin. Have the authors examined the leading-order nonlinearity? For the normal forms of the transcritical and pitchfork bifurcations, these are quadratic and cubic, respectively.

**Reply:** Thanks for the comments. The incoherent state does undergo a supercritical Pitchfork bifurcation when $ Re(\lambda_1)$ crosses zero. Since solving Eq. (12) gives rise to two new stable equilibria except for the unstable incoherent state at the bifurcation, $r_{1,2}>0$ in one equilibrium, and $r_{1,2}<0$ in the other. Due to the definition, $r_{1,2}<0$ is unrealistic though Eq.(12) allows for it. In Figure R_1 attached following, we show the equilibria acquired from Eq. (12) against $4\Delta/K $, which supports our claim.

Figure R_1: Bifurcation diagrams of $r_2$ (a) and $r_1$ (b) against $\Delta’=4\Delta/K$ obtained from Eq. (12). The partial synchronous states ($FP^{(3)}_1$ in wine and $FP^{(3)}_2$ in dark green) and incoherent state (in black) are stable (unstable) states denoted by solid (open) symbols. To support our claim that they are not transcritical bifurcations but pitchfork bifurcations, the unrealistic equilibria ($r_{1,2}<0$) are also shown. Across the pitchfork bifurcation (PB1), the incoherent state changes the stability. And the $FP^{(3)}_1$ ($FP^{(3)}_2$) disappear at the pitchfork bifurcation PB1(PB2) with increasing $\Delta’=4\Delta/K$. The parameters, $\beta=0.1$ and $\omega'_0=4\omega_0/K=1.5$, are same to Fig. 2(b) in the main text.

**Comment 3:** Third, the readability of the manuscript would be vastly improved if several large paragraphs were split into smaller paragraphs as I suggest below. I also include in the following list several other corrections.

- Please find a logical way to break the second paragraph of the introduction (lines 29-68) into smaller paragraphs.
- Line 141: Please confirm if you want "always not" or "not always" here. These have very different meanings.
- Line 150: till --> until
- Line 156: conjugated complex --> complex conjugate
- Line 162-3: the reference to the panels b and c in Fig. 2 is incorrect.
- The paragraph on page 7 is hard to read. It would be very helpful for the reader if this text is split into multiple paragraphs as follows:
  Line 161: begin a new paragraph with "Secondly, we consider...".
  Line 168: begin a new paragraph with "The third parameter...".
  Line 175: begin a new paragraph with "Using [the] above analysis, ...". Insert "the" as shown.
  Line 179: Join this text to the (new) preceding paragraph.
- In the first line of the caption of Figure 1, it would be helpful if "4\omega_0/K" were replaced with "\omega_0'=4\omega_0/K". This would help the reader connect the discussion in the text, which uses \omega_0', to the Figure. Please make similar adjustments to the other figures.

**Reply:** Thank you very much for these constructive comments. We have modified the manuscript by following these suggestions.

- Lines 182-183: Regarding the second point mentioned here, the unstable saddles that arise from curve SN2 have r1 != r2. That is why it was not reported in Martens et al. (2009); they only considered solutions with r1 = r2. Please clarify this point.

**Reply:** Thank you for the suggestion. We have clarified this point in the manuscript (lines 180-182).

- Line 184: Start a new paragraph at "In Fig 3(b)".
- Are the wiggly lines in the insets in Fig. 3 sample trajectories? These are confusing.

**Reply：**Yes, they are sample trajectories which show the trajectories from the same colored unstable fixed points. To make this point clear, we have added one sentence in the caption of Fig. 3 to clarify it.

- Line 189: The sentence that begins "Now it is clear...": This claim is not clear from looking at Figure 3.
- Line 192: New paragraph at "Compared with...".
- The contradictory statements in lines 198-200 are confusing. What are you trying to say? Please reword.
- Similarly, the sentences in lines 210-212 contradict each other. I don't understand what the authors want the reader to see in Figure 4.
- Figure 5 caption: change "sparse shadow region" to "shaded region".

**Reply：**We have reorganized these sentences or words to make them clear in the revised manuscript (for examples, lines 198-200, lines 211-212). Thank you very much for pointing them out.

We appreciate the help of Professor Mei Zhang from Department of Physics, Beijing Normal University. She helps us improve the language of the manuscript. We hope the English writing of the revised manuscript could meet the journal requirements.
