# Supplementary material for: Dynamics in the Sakaguchi-Kuramoto model with bimodal frequency distribution
Source: PLoS One. 2020 Dec 9;15(12):e0243196. doi: 10.1371/journal.pone.0243196 (PMC7725404; doi:10.1371/journal.pone.0243196)
Supplement: Attachment — Submitted filename: Response to Reviewers.doc [file pone.0243196.s002.doc]

**Manuscript No.:** PONE-D-20-12498R2
**Title:** Dynamics in the Sakaguchi-Kuramoto Model with bimodal frequency distribution

Dear Editor,

Thank you very much for handling our manuscript. We really appreciate the insightful comments from the Referee. We have revised the manuscript based on these comments. We hope this revised version could meet the publication standard of PLOS ONE.

For the revised submission, we would like to update our Funding Statement as follows: “This work is supported by National Natural Science Foundation of China under Grants No. 11575036 and No. 11805021, and BUPT Excellent Ph.D. Students Foundation under Grant No. CX2019138. The funders had no role in study design, data collection and analysis, decision to publish, or preparation of the manuscript.”

In the initial submission, we provided the funding statement in the manuscript but did not state them in the Funding section of the online submission. We must apologize for our carelessness. We would be grateful if you could help us update the financial disclosure statement.

**The followings are the details of the reply.**

**Reviewer #1:** The authors have addressed my concerns. Ambiguities have been removed and the English is now sufficiently intelligible.

R**eviewer #2:** The authors of the manuscript “Dynamics in the Sakaguchi-Kuramoto Model with bimodal frequency distribution” investigate the dynamics of a population of phase oscillators whose natural frequency terms are drawn from a bimodal distribution. The coupling between oscillators is global and occurs via the sine of their pairwise phase differences. A phase lag parameter, or frustration parameter, is added, which leads to a bimodal Kuramoto-Sakaguchi model. The model in this particular form and with respect to asymmetric frequency distributions has not been analyzed in the literature, yet. Moreover, the authors report the “revival” of the incoherent state: when increasing a bifurcation parameter, the stable incoherent state first becomes unstable and later on stabilizes again. The collective behavior of the system is studied following the Ott-Antonsen ansatz, which allows for deriving analytically tractable low-dimensional dynamics of the Kuramoto order parameter. Here, the system consists of three coupled ordinary differential equations, whose bifurcation structure is investigated with analytic and numerical techniques.

**Comment 1:** Although the here-reported form of the Kuramoto-Skaguchi model is new, the mathematically sound results for the symmetric case, which is central to this manuscript, can be anticipated from Martens, Bick and Panaggio (Chaos 26, 094819, 2016) and Refs. 20, 29, when taking into account that there is an equivalence between the bimodal formulation and a two-coupled-population formulation of coupled phase oscillators as established, e.g., in Ref. 30. The manuscript can present an important additional contribution to the field if, in a revised version, the authors can explicate whether the phase lag parameter \beta and the heterogeneity parameter \Delta have differential effects on the collective dynamics.

**Reply:** Thanks for the comment. Following the suggestions, we have made a brief discussion on the effects of \Delta and \beta on collective dynamics in the revised manuscript at lines 78-88 on page 3.

**Comment 2:** The novelty of this manuscript is the impact of the asymmetric bimodal distribution on the revival of the incoherent state, which unfortunately is not yet worked out thoroughly. Since similar revival results have been obtained by Omelchenko and Wolfrum in for unimodal frequency distributions, the manuscript can significantly be improved when the authors discuss in more detail the differences and similarities with their work and that by Omelchenko and Wolfrum.

**Reply:** Thank you very much for the suggestion. In the revised manuscript, we have discussed in more detail the differences and similarities with the work by Omelchenko and Wolfrum at lines 276-286 on pages 9.

**Comment 3:** As to the presentation of results, the authors should clarify why unstable solutions and their bifurcations (as in Figs. 1, 2, 3 and 4) are studied extensively. As their numerical analysis attains large attention through Figs. 1 and 2, the reader is misled in that unstable solutions seem to be crucial. But I doubt that they have any real effect on the dynamics of the population activity [apart from the SN2 curve in Fig. 3b between the CP and TB points].

**Reply:** We agree with that unstable solutions have no any effect on the long-term dynamics of the population dynamics. However, the existence of unstable solutions greatly shapes the topological structure of the underlying phase space and has strong impacts on the transient dynamics of the model. Moreover, these unstable solutions might become stable with the change of parameter and take effects on the long-term dynamics of the model. Therefore, in the perspective of stability analysis, the exploration of unstable solutions is necessary. We have explained it at lines 193-199 on pages 7.

**Comment 4:** The presentation of the resulting dynamical regimes appears unclear: why are the sub-order parameters r_1 and r_2 the important properties? The insets in Fig. 3 suggest that r_1 and r_2 really are the central properties, but why they are of interest, does not become clear. And why is not the global order parameter Z characteristic for the network dynamics?

**Reply:** Thanks for the comment. Actually, the network dynamics can be reflected by the global order parameter Z. If we just numerically simulate the coupled phase oscillators Eqs. (1,2), Z will be the candidate for exploring the population dynamics. However, in this work, we study the population dynamics by considering the low-dimensional reduced model, deduced based on the OA ansatz, in which the sub-order parameters r_1, r_2, and psi are the state variables. Consequently, we pay attention to r_1, r_2, and \psi to explore the population dynamics. We have emphasized this point at the lines 112-114 on page 4.

**Comment 5:** The insets in Fig. 3 are hard to understand. A more intuitive explanation, and/or schematics of the global states, would be helpful for the reader. Maybe the layout of inset figures can be changed to that used in Bick, Martens and Panaggio (Chaos 2016, Chaos 2018).

**Reply:** Thanks for the comment. We have rewritten the figure caption in Fig. 3 and try to make it to be readable. We also follow the suggestions and have updated Fig. 3 by adding arrows to the trajectories of evolution. The solutions and trajectories on the plane of r_1 and r_2 could be distinguished. [W](../../../../D:/install/Youdao/Dict/8.9.4.0/resultui/html/index.html#/javascript:;)e [hope](../../../../D:/install/Youdao/Dict/8.9.4.0/resultui/html/index.html#/javascript:;) the modifications are helpful for the reader. Besides, we have included the two related works in the references (Refs. [21], [34]).

**Comment 6:** I don’t understand what Fig. 4 adds to the manuscript. If the purpose is to highlight how beta changes the bifurcation diagram, then there are at least two better options:
a. Add more bifurcation diagrams similar to Fig. 3 for different choices of beta.
b. Make Fig. 3b 3-dimensional by adding a beta-axis.

**Reply:** As we have worked out, the phase lag \beta impacts on the dynamics in a non-monotonic way at large \omega_0. For example, when \beta increase from zero to \pi/2, the incoherent state may be unstable for intermediate \beta and stay stable otherwise. To figure out the non-monotomic effects of \beta on the model dynamics, we presented the bifurcation diagrams on 4\Delta/K-\beta plane.

To make our motivation clear, we have written the paragraph at lines 225-229 and 231-233 on pages 7 and 8 in the revised manuscript.

**Comment 7:** Line 220: Please start a new subsection in order to distinguish the parts using a symmetric versus an asymmetric distribution, respectively.

**Reply:** Thank you for the suggestion. We have modified the manuscript by adding new subsections on pages 4 and 8.

**Comment 8:** In Fig. 5c, \omega_0 takes on negative values, but since it is considered to be half the distance between the peaks of the bimodal frequency distribution, how can it be negative? Please clarify.

**Reply:** Thanks for the comment. In the case of the asymmetrical bimodal frequency distribution where different peaks have different widths, \omega_0 is defined as \omega_1-\omega_2 where \omega_1 is center frequency of the peak with width \Delta_1 while \omega_2 for the peak with width \Delta_2. Therefore, \omega_0<0 means that the peak frequency \omega_1<\omega_2 while \omega_0>0 means that the peak frequency \omega_1>\omega_2. These two situations are not the same. In this revised manuscript, we have clarified it at lines 252-255 on page 8.

**Comment 9:** In the Conclusion, line 257, please enumerate here your main findings in which way (how) beta affects the dynamics. Moreover, it would helpful to show whether beta has any differential effects compared to the other important parameter \Delta because, in general, both prevent the system to fully synchronize. In line 211 the authors already point to the counter-intuitive phenomenon that close to 4\Delta = K, a larger frustration parameter \beta “tend[s] to destabilize the incoherent state”, but the mechanism why this is so remains unclear.

**Reply:**

1. “In the Conclusion, line 257, please enumerate here your main findings in which way (how) beta affects the dynamics.”

We have followed the suggestion and revised the conclusion at lines 294-297 on page 9.

2. “Moreover, it would helpful to show whether beta has any differential effects compared to the other important parameter \Delta because, in general, both prevent the system to fully synchronize.”

This comment is similar to comment 1, we have presented a comparison between \beta and \Delta on model dynamics at at lines 78-88 on page 3.

3.“In line 211 the authors already point to the counter-intuitive phenomenon that close to 4\Delta = K, a larger frustration parameter \beta “tend[s] to destabilize the incoherent state”, but the mechanism why this is so remains unclear.“

The comment is similar to comment 6. As presented in the reply to comment 6, we have rewritten the corresponding contend to make our points clear.

**Comment 10:** Line 259: what are these conditions for better observing the revival? Please write them out here explicitly and, perhaps, the authors can also elaborate here on their hypothesis in lines 248/249.

**Reply:** Thank you very much for the suggestion. We have phrased the conditions for the revival of incoherent state at lines 276-286 on pages 9 in the revised manuscript.
